# Right-sided ureteral hemangiosarcoma in a paraplegic dog

**DOI:** 10.1186/s12917-024-04114-8

**Published:** 2024-06-22

**Authors:** Suellen Rodrigues Maia, Mayara Manochio, Lara Vilela Soares, Yury Carantino Costa Andrade, Alef Winter Oliveira Alvarenga, Leandro Zuccolotto Crivellenti

**Affiliations:** 1 School of Veterinary Medicine and Animal Science (FMVZ), Universidade Estadual Paulista (UNESP), Botucatu, São Paulo, Brazil; 2 Self-employed veterinarian, Franca, São Paulo, Brazil; 3 Graduate Program in Veterinary Science (PPGCV), College of Veterinary Medicine (FAMEV), Universidade Federal de Uberlândia (UFU), Uberlândia, Minas Gerais Brazil; 4 Unidade Integrada de Veterinária (UNIVET), Ribeirão Preto, São Paulo, Brazil

**Keywords:** Ureteral obstruction, Pyelography, Hydronephrosis, Ureteral neoplasia

## Abstract

**Background:**

This study aims to describe a rare case of primary ureteral hemangiosarcoma, in which surgical intervention preserved the kidney and ureter after tumor removal.

**Case presentation:**

A 13-year-old, neutered male dog, weighing 14 kg, mixed-breed, presented with apathy, anorexia, acute-onset vomiting, and abdominal discomfort during the physical examination. Ultrasonography and pyelography revealed a right-sided dilation of the renal pelvis and ureter due to complete obstruction in the middle third of the ureter. A mass obstructing the lumen of the right ureter was completely resected, and ureteral suturing was performed, preserving the integrity of the involved structures. Histopathology confirmed primary ureteral hemangiosarcoma. Due to the local and non-invasive nature of the mass, chemotherapy was not initiated. The patient’s survival was approximately two years, and normal renal function was preserved throughout this period.

**Conclusions:**

Considering this type of tumor in the differential diagnosis of upper urinary tract obstructive disorders. Furthermore, the preservation of the ureter and kidney is a suitable therapeutic option after surgical resection of non-invasive tumors.

## Background

Hemangiosarcoma is a rapidly growing and aggressive malignant neoplasm, demonstrating significant metastatic potential due to its vascular association [1]. In dogs, the primary site of development is typically the spleen, followed by other locations such as subcutaneous tissue, dermis, liver, and right atrium [2–5]. Less common sites include the retroperitoneum, reproductive system, lung, and urinary system [6–9].

Primary neoplasms in the ureteral region are rare in dogs, and most often benign, including fibropapillomas, fibroepithelial polyps, and transitional cell papillomas [10–12]. Limited reports in dogs describe the occurrence of malignant neoplasms in this region, such as leiomyosarcomas [13, 14] and transitional cell carcinomas [15]. Even more rarely, only two cases of ureteral hemangiosarcoma have been reported to date [16, 17]. Given the rarity of this presentation, this report aims to provide detailed insights into the occurrence of ureteral hemangiosarcoma in an elderly dog, encompassing clinical and pathological manifestations, surgical treatment, diagnosis, and survival. This contributes evidence to the literature, emphasizing the importance of considering this type of tumor in the differential diagnosis of upper urinary tract disorders. To the best of our knowledge, this is the third reported case of primary ureteral hemangiosarcoma and the first to preserve the ureter and kidney without subsequent recurrence.

### Case report

A male mixed-breed dog, 13 years old, weighing 14 kg, neutered, with a history of paraplegia due to an old traumatic injury (occurring when the animal was a puppy), presented with acute onset apathy, hematuria, anorexia, and vomiting. Due to the animal’s neurological condition, the dog’s urination history involved the need for manual bladder compressions throughout the day to achieve minimally acceptable bladder emptying, performed by its owner since the onset of its paraplegic condition. The patient also had a history of recurrent urinary tract infections due to chronic neurological bladder dysfunction. Pronounced abdominal discomfort was noted upon physical examination. Laboratory findings revealed mild azotemia in the serum biochemistry (urea 84 mg/dL and creatinine 2.0 mg/dL), with no other abnormalities in either biochemical analyses or the complete blood count.

**Fig. 1:**
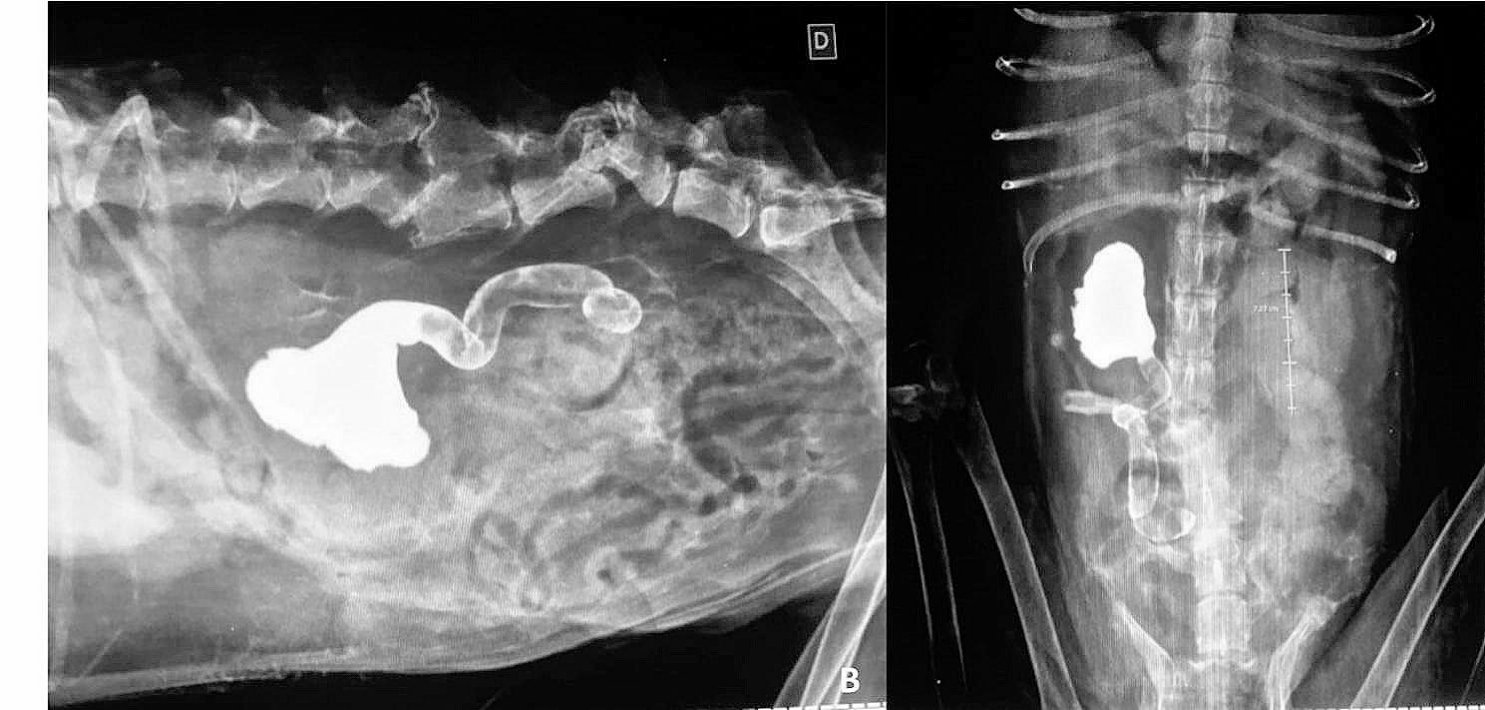
Ultrasound-guided percutaneous anterograde pyelography, in lateral (**A**) and ventrodorsal (**B**) projections. Radiographs were obtained immediately after injection of iodinated contrast, noting dilation of the right renal pelvis and ureter, obstruction of the contrast column in the middle third of the right ureter, as well as deviation of the bony axis of the lumbar spine, luxation of L3-L4, L5-L6, bone remodeling of L6 and articular processes of L3 and L4, due to a history of old trauma. Radiographic images were obtained after the administration of iodinated contrast at a dose of 600 mg/kg

Ultrasonographic examination disclosed hydronephrosis in the right kidney and hydroureter extending to the middle third, originating from a likely point of obstruction; however, the reason possibly associated with the obstruction was not determined by this evaluation. A pyelogram was performed to delineate the involved structures and precisely identify the obstructive site for surgical planning. The contrast examination consisted of the percutaneous administration of iodinated contrast, at a dose of 600 mg/kg, directly into the right renal pelvis with the assistance of ultrasound (the volume of contrast applied was equivalent to the volume removed by pyelocentesis). This confirmed a complete obstruction in the middle third of the right ureter, with no contrast progression beyond the identified point (Fig. 1).

**Fig. 2:**
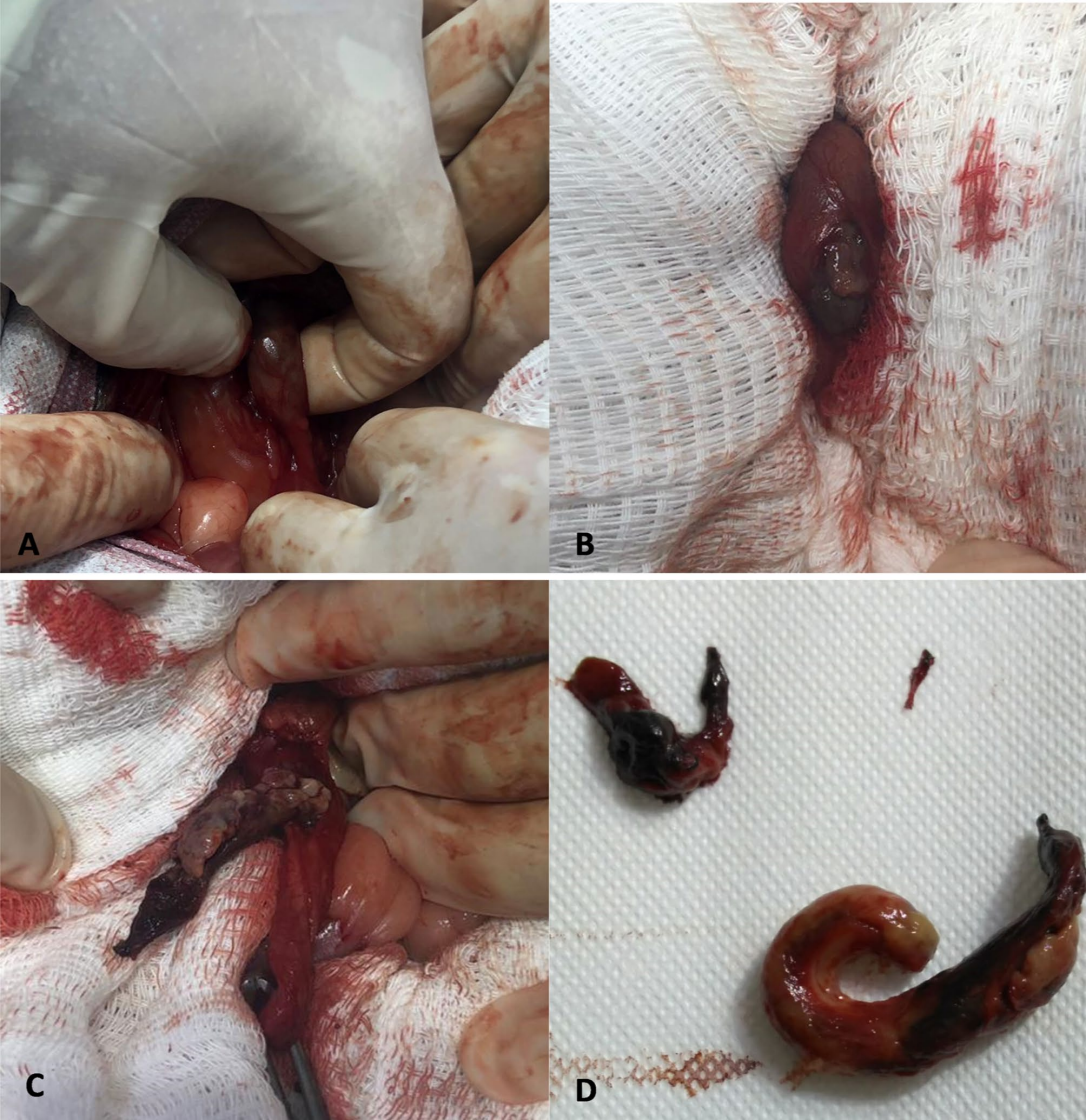
Surgical Procedure. **A**) Identification of the affected ureter and isolation of the obstruction site (region between the surgeon’s fingers). **B**) Longitudinal incision of the ureter and appearance of the tissue obliterating the lumen. **C**) Dissection and exposure of the mass within the ureter lumen. **D**) Final appearance of the mass after complete removal. Only one mass was found and removed (fragmentation of the mass occurred after its manipulation)

After laparotomy, the right ureter was identified, isolating the previously revealed obstruction point from the imaging examination (Fig. 2A). A longitudinal incision was made at the affected site, revealing tissue content causing obliteration of the ureteral lumen (Fig. 2B). The mass was fully exposed, demonstrating weak adhesion to the ventral wall of the ureter only in a small portion (approximately 0.5 mm) (Fig. 2C), and subsequently easily resected and removed from the ureteral lumen, along with the small portion of the involved ureter (Fig. 2D). The absence of neoformations in other regions of the urinary tract, as well as in abdominal organs, suggested a possible primary ureteral neoplasm. The ureteral incision was sutured using simple interrupted sutures with absorbable suture (poliglecaprone 25, 6 − 0), preserving the patient’s upper urinary tract, followed by routine closure of the abdominal cavity. The surgery was completed in 1 h and 40 min.

There were no complications during the surgical/anesthetic procedure, and the animal was discharged with follow-up care.

**Fig. 3:**
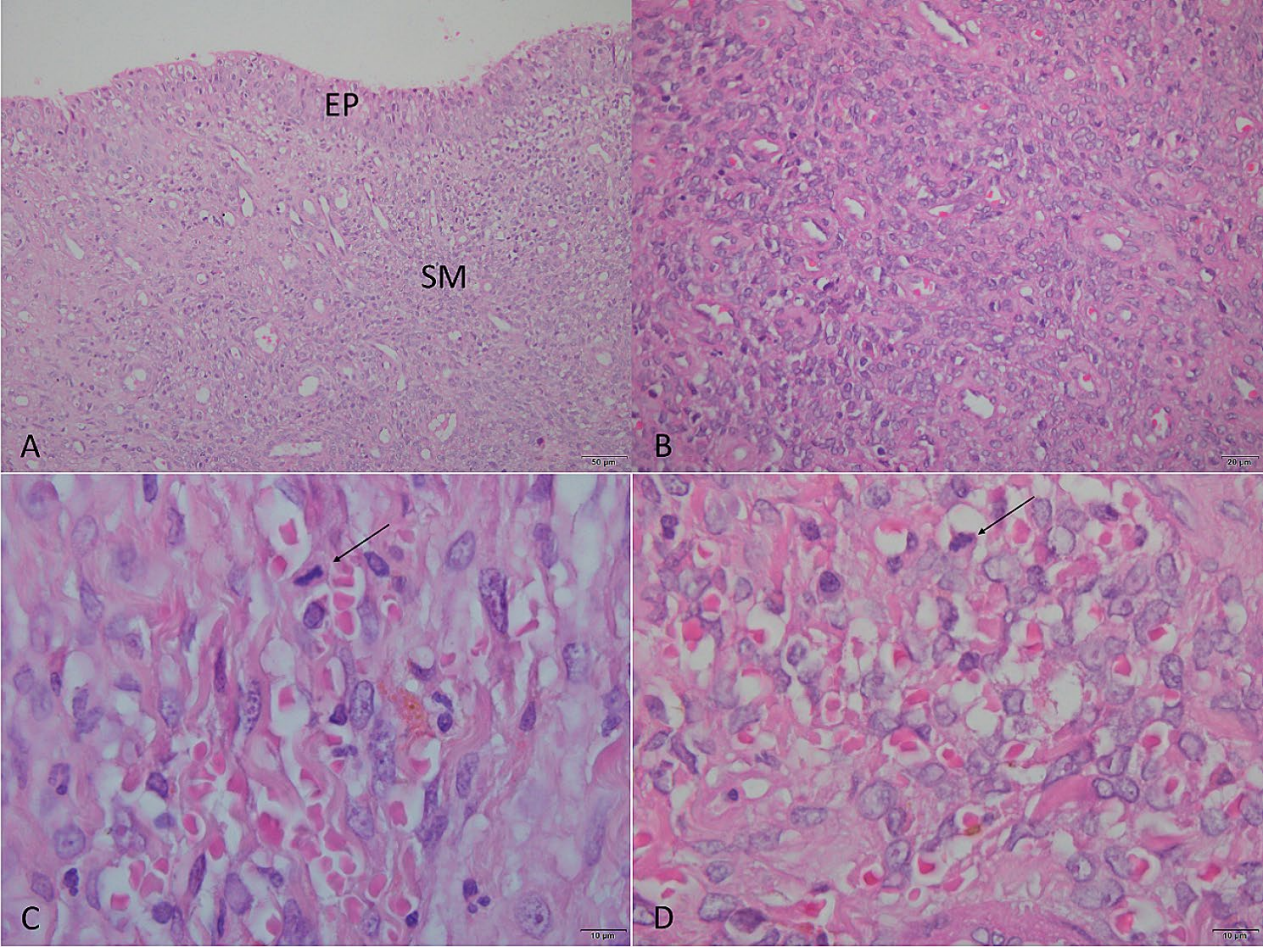
Histopathological Findings. **A**) Fragment lined by well-differentiated urothelial epithelium (EP). Submucosa (SM) with diffuse malignant mesenchymal neoplasia. Hematoxylin and Eosin (HE) staining. Magnification 200x. **B**) Neoplastic cells with solid and diffuse organization. HE staining. Magnification 400x. **C**) Mitotic figure, cell in metaphase (arrow). HE staining. Magnification 1,000x. **D**) Malignant mesenchymal proliferation with the formation of irregular vascular spaces filled with erythrocytes. Neoplastic cells exhibit pleomorphic nuclei with vesicular appearance and prominent nucleoli. Mitotic figure, cells in prophase (arrow). HE staining. Magnification 1,000x

The histopathological examination of the mass confirmed the neoplastic nature of the removed tissue (Fig. 3), revealing a neoplastic proliferation composed of endothelial cells, mimicking disorganized vascular formations. Elongated cells with pleomorphic nuclei and prominent nucleoli, mild mitotic activity, and extensive areas of necrosis and hemorrhage were observed upon evaluation. On the surface of the mass, well-differentiated urothelial epithelium composed of 3 to 6 layers of cells was present. Based on these characteristics, the final diagnosis was consistent with ureteral hemangiosarcoma.

Following the diagnosis, thoracic radiographs were obtained, along with postoperative ultrasound monitoring, which did not reveal any changes consistent with metastases. Four months post-surgical procedure, the animal exhibited overall well-being, alertness, normal eating habits, and its usual behavior. There was complete reversal of hydronephrosis and hydroureter on ultrasound, and serum biochemistry assessment revealed a return of urea and creatinine levels to within the reference range (44 mg/dL and 0.6 mg/dL, respectively). The animal demonstrated a survival period of two years, maintaining a good quality of life and overall well-being. However, due to an acute and severe presentation of respiratory alterations apparently unrelated to metastases (thoracic radiographs did not show any changes that justified the clinical presentation), the patient deteriorated despite supportive treatment. Consequently, euthanasia was chosen by the owner.

## Discussion and conclusions

Hemangiosarcoma is a malignant mesenchymal neoplasm that can be classified into visceral and non-visceral types based on its location [18]. Primary occurrence of this type of tumor in the canine ureter is rare [16, 17], as are other types of tumors in this region [19–23]. In dogs, only two reports describe primary ureteral hemangiosarcoma [16, 17]. In one of the cases, metastases developed shortly after tumor resection [16], differing from the presentation reported here. However, it is important to highlight that due to the acute worsening and respiratory evolution presented by the dog in this report, the possibility of pulmonary thromboembolism, together with metastases that may not have been radiographically identifiable, cannot be ruled out. In any case, despite being a highly metastatic tumor, the timing of metastatic presentations can be variable, as indicated by this and other cases described.

In contrast to previously reported cases [16, 17], the affected dog in this report was male but was of a similar age range. Considering the overall occurrence of visceral hemangiosarcoma in dogs, those over 10 years old are generally more affected [21], aligning with the occurrence of primary ureteral hemangiosarcoma in this case.

The clinical signs presented in this report, including abdominal pain upon palpation, apathy, hematuria, anorexia, and vomiting, although nonspecific, resemble cases of malignant ureteral tumors previously reported, such as leiomyosarcoma [14], sarcoma [22], and urothelial carcinoma [23]. Renal dysfunction secondary to the condition or increased pressure in the renal capsule due to hydronephrosis may explain the observed clinical signs, as well as the presence of the tumor itself, considering that signs usually associated with visceral hemangiosarcoma are also vague and nonspecific [1]. It’s is noteworthy that specific signs of urinary tract involvement, such as hematuria, might not necessarily be present, as observed in the case described by Polit et al. [17].

The prolonged duration of paraplegia and recurrent urinary tract infections observed in the canine patient described here prompts discussion regarding the possibility of a relationship between these comorbidities and neoplastic condition. Although such presentations are not commonly described in veterinary literature, human studies have investigated the link between urinary tract neoplasms, particularly involving the bladder, in patients with spinal cord injuries [24–27]. The main risk factors in these cases include chronic urinary drainage using catheters, recurrent urinary infections, and a history of urolithiasis [28, 29]. While definitive cause-and-effect relationships cannot be presumed, it is important to consider that paraplegic patients who are chronically managed to maintain urinary flow require increased attention to the occurrence of tumors involving the urinary tract.

In this report, ultrasonographic and contrast radiographic images revealed hydronephrosis and hydroureter affecting the right kidney and ureter, respectively, confirming complete ureteral obstruction in the middle third. This finding differs in location from previous case reports of the same tumor, which indicated tumors in the distal ureter [17] and proximal ureter [16]. According to literature on dogs, the location of the tumor along the ureteral length can be related to the malignancy of the neoplasm, with distal tumors generally being malignant, while proximal tumors tend to be benign [17, 22, 30]. However, although information regarding the location of the ureteral tumor and its malignancy is described, due to the lack of existing evidence, it is not possible to attribute characteristics of malignancy based on tumor location at this time. Just as described here and corroborating with Troiano and Zarelli [16], the occurrence of primary ureteral hemangiosarcoma can affect any portion of the ureteral extension.

According to literature on dogs, the location of the tumor along the ureteral length can be related to the malignancy of the neoplasm, with distal tumors generally being malignant, while proximal tumors tend to be benign [17, 22, 30].

However, as described here and corroborating with Troiano and Zarelli [16], the occurrence of primary ureteral hemangiosarcoma does not adhere strictly to this premise, revealing that any portion of the ureteral extension can be subject to this type of tumor.

Furthermore, this report differs from two others involving the same type of tumor [16, 17] due to its non-invasive local nature, being easily resectable from the ureter and not presenting significant adhesions. This allowed for the preservation of the affected kidney and ureter. The two dogs affected by ureteral hemangiosarcoma previously described had radical surgical approaches as the treatment of choice. In one case, left nephrectomy and ureterectomy were performed, while in the other, left ureteronephrectomy, partial cystectomy were performed [16, 17]. While many factors are involved in the surgical planning for each specific case, the majority of reports involving ureteral neoplasms in dogs resulted in ureterectomy and nephrectomy [13–17, 22, 23], making this the first account in which these noble structures were preserved.

It is essential to emphasize that preserving nephrons should always be the central goal of any surgical therapeutic approach to the urinary tract. This is because, despite imaging changes, it is not possible to estimate the residual functionality of the affected organ. The patient in this report was azotemic before the surgical procedure, indicating that the compensatory capacity of the contralateral kidney was also impaired. The return of urea and creatinine values for this patient to species reference ranges after the procedure reflects the impact on the functional reserve of the previously affected kidney. Similarly, in humans, although upper urinary tract tumors are also rare [31–33], it was reported that malignant ureteral tumors can be cured by local resection [34–36].

Adding to previously published data [16, 17], where a survival of 40 days and a follow-up of 120 days were reported, the two-year survival along with the quality of life of the dog in this report demonstrates the variety of possible outcomes for this type of presentation. Due to the rarity of primary ureteral hemangiosarcoma in dogs, prognostic information is scarce and precludes any determinations regarding our results or predictions of outcomes.

Based on the data described here, it emphasizes the importance of including primary ureteral hemangiosarcoma in the differential diagnoses of obstructive conditions in the ureter of dogs, whether are associated with clinical signs of the urinary tract or metastases or not.

A possible association in dogs with spinal cord injuries is unclear. Preservation of affected urinary tract structures should be prioritized, especially when possible contralateral renal dysfunction is ongoing. Prognoses cannot yet be definitively determined due to the rarity of this tumor type.

## Data Availability

No datasets were generated or analysed during the current study.

## Abbreviations

EP: Epithelium
SM: Submucosa
HE: Hematoxylin and Eosin
